# Personal

**Published:** 1889-07

**Authors:** 


### Personal.

DR. WILLIAM A. WHEELER, U. S. Marine Hospital Service, who for over four years had the surgical charge of the marines in this city, is now stationed at Norfolk, Va. We regret that the demands of the service called him to other fields of labor, for, besides being a genial and cultured gentleman, he is a most accomplished surgeon, and filled the position here with marked ability. We note with pleasure his success as a teacher. For two years the Chair of Surgery in the Medical Department of Niagara University was filled by Dr. Wheeler, and his clear and logical teachings were warmly appreciated by both faculty and students. The profession in Virginia gains a valued member by this transfer, and we bespeak for him the generous welcome which that warm-hearted and hospitable people always tender to accomplished gentlemen. Dr. Devan has been transferred to this city to take the position vacated by Dr. Wheeler. We cordially welcome the new incumbent to our city, and trust the high position occupied by his predecessor will be attained by him.

### *PERSONAL.*

DR. C. C. FREDERICK, of this city, has been appointed a member of the Board of Examiners in Midwifery, for Erie county, vice Dr. J. W. Keene, removed to California. This will be recognized as a most excellent appointment, as Dr. Frederick is thoroughly qualified for the place.

